# Reproducibility and Validity of the Kerlan-Jobe Orthopedic Clinic Shoulder and Elbow Score (Japanese Version)

**DOI:** 10.7759/cureus.59139

**Published:** 2024-04-27

**Authors:** Kaoru Sasagawa, Masashi Kawabata, Norifumi Takaki, Masaaki Tsuruike, Todd S Ellenbecker, Yusuke Tsuihiji, Hiroyuki Watanabe, Naonobu Takahira, Norikazu Hirose

**Affiliations:** 1 Therapeutics, Medical Plaza Ichikawa Station, Ichikawa, JPN; 2 Therapeutics, School of Allied Health Sciences, Kitasato University, Sagamihara, JPN; 3 Therapeutics, Blueprint Performance Lab, Saitama, JPN; 4 Therapeutics, Water Holder Co., Tokyo, JPN; 5 Therapeutics, Banner Sports Medicine, Scottsdale, USA; 6 Therapeutics, Kitasato University Graduate School of Medical Sciences, Sagamihara, JPN; 7 Orthopaedic Surgery, School of Allied Health Sciences, Kitasato University, Sagamihara, JPN; 8 Therapeutics, Faculty of Sport Sciences, Waseda University, Saitama, JPN

**Keywords:** patient-reported outcomes, overhead athlete, elbow injury, shoulder injury, q-dash, j-kjoc, kerlan–jobe orthopedic clinic shoulder and elbow

## Abstract

Background: The Kerlan-Jobe Orthopedic Clinic (KJOC) questionnaire is a self-reported performance and functional assessment tool with good reliability and validity for overhead athletes with shoulder and elbow injuries. This study aimed to develop a Japanese version of the KJOC (J-KJOC) to clarify its reproducibility and validity for use by Japanese university baseball players.

Methods: The J-KJOC was translated according to the guidelines for cross-cultural adaptation. A total of 88 university baseball players completed the J-KJOC and the Quick-Disabilities of the Arm, Shoulder, and Hand (Q-DASH) questionnaires. Thirty players completed the J-KJOC two times after a median interval of two weeks. We assessed the absolute reliability, construct validity, internal consistency, and test-retest reliability.

Results: Cronbach's alpha coefficients ranged from 0.88 and the intraclass correlation coefficient for the total score was 0.91. A fixed bias was absent in the J-KJOC scores (mean difference: −2.2, 95% CI: −4.8 to 0.5). Furthermore, the J-KJOC score was correlated with the Q-DASH-disability/symptom (r = −0.60, p<0.01) and Q-DASH-sports/music (r = −0.63, p<0.01) scores but not correlated with the Q-DASH-work score (r = −0.11, p = 0.316).

Conclusions: The J-KJOC questionnaire demonstrated good reproducibility and validity for assessing upper arm performance in Japanese university baseball players. The results of this study support the use of the J-KJOC for Japanese-speaking baseball players. Further research using this instrument on other types of overhead athletes is needed to determine its wider utility in sports medicine applications.

## Introduction

Shoulder and elbow pain and injuries are common in overhead sports. Overhead activities, such as throwing and serving, impose significant stress on the shoulder and elbow [1,2], and the repetition of these stresses can lead to injuries [3]. The incidence of injury in baseball pitchers resulting from repetitive throwing is 3.5 times higher than that in healthy baseball pitchers [4]. Moreover, an injury progressing to a point requiring surgery can substantially impact the player's career. Professional tennis players who underwent arthroscopic shoulder surgery took an average of 279 days to resume playing, and their world ranking dropped for up to two years after the surgery, indicating a significant career impact [5]. Limited joint range of motion [6], muscle weakness [7], and scapular dyskinesis [8] are common physical dysfunctions that can contribute to injuries during athletic activities [9]. However, not all athletes with these dysfunctions necessarily develop injuries. Some reports have documented cases in which athletes with limited joint range of motion and scapular dyskinesis did not experience injuries [10,11], suggesting limitations in using specific dysfunctions as definitive outcomes. Therefore, early detection of injury in overhead sports athletes is crucial for injury prevention and identifying the need for more comprehensive evaluations of dysfunction, including closer monitoring of the number of games, training intensity, and subjective complaints from the athlete.

Traditionally, the evaluation of upper limb function and performance in athletes has relied on measures such as range of motion and muscle strength [12]. However, researchers have emphasized the use of patient-reported outcomes (PROs) to focus on the symptoms experienced by patients and athletes to develop appropriate treatments [13]. The Disabilities of the Arm, Shoulder, and Hand (DASH) questionnaire [14] and its shortened version, i.e., the Quick DASH (Q-DASH) questionnaire, are two of the commonly used PROs for upper limb assessments [15]. However, these questionnaires are not specific to athletes and may inadequately capture the functional status and performance of their upper limbs. In Japan, similar evaluation methods are often employed; however, an assessment tool that encompasses not only postoperative pain and joint function but also pitching performance is warranted for postoperative evaluation and injury prevention in athletes.

The Kerlan-Jobe Orthopedic Clinic (KJOC) questionnaire is a sport-specific questionnaire that evaluates the upper limb performance of overhead athletes. The KJOC score reflects small changes in athletes’ upper limb function and performance sensitively [16]. It is characterized by PROs and consists of 10 items, with a maximum score of 100 points. The KJOC has been used primarily for postoperative evaluation [17] and as a scale for injury prevention [18] in overhead sports. Healthy baseball players typically score ≥90 points on the KJOC [19], whereas healthy swimmers score ≥85 points [20]; lower scores may increase the risk of disability. Another notable feature is its sensitivity to shoulder and elbow dysfunction, as well as minor changes in performance [16]. The KJOC has been extensively used in English-speaking countries, with demonstrated reliability and validity [16]. The translated versions have been validated in several languages [21,22]. However, its reliability and validity in the Japanese population have not yet been reported.

Japan has one of the largest baseball player populations worldwide, with many players competing at high levels. A substantial number of players suffer from injuries [23]. Developing a Japanese version of the KJOC (J-KJOC) and confirming its reliability and validity would have meaningful implications for Japanese athletes and the worldwide dissemination of information. We hypothesized that the J-KJOC would be a valid, reliable, and responsive tool to assess shoulder and elbow functionality in Japanese overhead athletes.

## Materials and methods

Translation and cross-cultural adaptation

For preparing the J-KJOC, we followed the guidelines for cross-cultural adaptation [24]. The original version of the KJOC questionnaire was first translated independently by two healthcare professionals (certified athletic trainers). These two professionals compared the two versions of the questionnaire and discussed inconsistencies until a consensus was achieved. Following the guidelines, the Japanese version was translated back into English by an English-Japanese bilingual (see Appendix A); subsequently, the back-translated version was reviewed by an expert in this research area who is a native English speaker and was carefully revised several times. In July 2023, the development of the final J-KJOC was completed.

Assessment tools

The KJOC is an athlete-specific functional and performance assessment of the shoulder and elbow consisting of two sections and 10 questions with a visual analog scale on a 10-cm line. The left and right ends of the scale have a score of 0 and 10, respectively. The higher the score, the better the shoulder and elbow function. The scores were measured using a ruler from the left end to the mark created by the respondent and were recorded to one decimal place. The overall KJOC score ranges from 0 to 100, with 100 indicating the best shoulder and elbow conditions. Moreover, the tool comprises five subsections about the current status of upper extremity injury, three about the current level of competition, and three about the impact of the injury on play [16].

The Q-DASH-disability/symptom comprises 11 items for assessing physical function and symptoms in the upper limbs (score: 0 denotes best, 100 denotes worst). The Q-DASH consists of two optional modules, namely the work module (Q-DASH-work, four items) for workers with a high degree of physical performance and the sports/music module (Q-DASH-sports/music, four items) for athletes or performing artists. The score cannot be calculated if more than one item is missing. An optional module score may also not be calculated if an item is missing.

Participants and ethical considerations

We enrolled 88 collegiate baseball players in Japan (age: 20.4 SD, 1.3 years; career: 11.6 SD, 2.8 years; fielders: 66; pitchers: 20), with Japanese as their first language. The inclusion criteria were collegiate baseball players who practice at least twice a week. The exclusion included a history of neuropathic pain, trauma, or surgery of the upper limb. No participant was excluded in this study. The Ethics Committee of Kitasato University approved this study (approval no. 2022-032-3). All participants provided written informed consent before participation.

Data collection

The questionnaires were administered at two time points from September to December 2023 at two university baseball clubs engaged in active training and competition. All 88 players were instructed to complete printed versions of the J-KJOC and Q-DASH to assess construct validity. The J-KJOC questionnaire was redistributed within four weeks to assess the test-retest reliability between the two time points (30 collegiate baseball players).

Sample size calculation

The sample size was calculated by an a priori power analysis using G*Power version 3.1 (Heinrich Heine University Düsseldorf, Düsseldorf, DEU) [25]. Based on an α of 0.05, a power of 0.80, the correlation coefficient of 0.72 [21], and the intraclass correlation coefficient (ICC) of 0.505 to 0.937 [21] with respect to the results of a previous study, size analysis indicated that a total sample size of 10 and 26 would be required to demonstrate a significant difference in the correlation coefficient and ICC, respectively.

Statistical analysis

The construct validity of the J-KJOC was assessed by the correlations between the J-KJOC score and scores of the Q-DASH modules of disability/symptom, work, and sports/music, which were determined using the Spearman correlation coefficients and their corresponding p-values. The internal consistency of the J-KJOC reliability was evaluated using Cronbach’s alpha. The Cronbach's alpha coefficients ranged from 0 to 1, with a value of 0.7 indicating internal consistency [26]. The test-retest reliability was determined using the ICC (1, 1) and the corresponding 95% CI. Good correlation was defined as excellent reliability with coefficients ranging from 0.75 to 1.00, good reliability with coefficients ranging from 0.60 to 0.74, fair reliability with coefficients ranging from 0.40 to 0.59, and poor reliability with coefficients <0.40 [27]. The standard errors of the mean (SEM) and minimal detectable change (MDC) were estimated using the following formulas [28]: SEM=SD×(√1-ICC) and MDC=SEM×1.96×√2n.

The Bland-Altman plot was created by plotting differences against the mean between the test and retest to assess absolute reliability [29]. A fixed bias was identified when the 95% CI for the mean difference did not include zero. The 95% CI for the lower and upper limits of agreement were calculated using the following formula [29]: (mean ±1.96 ×SD) ± t ×√(3SD2/n) where 'mean' is the mean difference between the test and retest, 'SD' is the standard deviation of the difference, and 'n' is the sample size. All statistical analyses were performed using EZR (Saitama Medical Center, Jichi Medical University, Saitama, JPN) [30], which is used in R software (RStudio, Boston, MA, USA). More precisely, it is a modified version of the R Commander designed to add statistical functions frequently used in biostatistics. The significant level was set at α = 0.05.

## Results

Translation and cross-cultural adaptation of the KJOC

In the original version of the KJOC, the questionnaire asked participants to select their current performance level from the following options: professional major league, professional minor league, intercollegiate, and high school. On the other hand, in Japan, there are two categories between the minor league (professional second league) and the intercollegiate, namely the independent league and the Shakaijin league, as a semi-professional league. Thus, we added these two leagues to the options in the J-KJOC.

Participant characteristics

Table 1 and Table 2 summarize the participant characteristics (n = 88) and mean values of the J-KJOC and Q-DASH scores.

**Table 1:** Participant characteristics (n=88)

Characteristics	No. of players (%)	Mean (SD)	Range
Age (years)		20.4 (1.3)	18.0–23.0
Career (years)		11.6 (2.8)	5–18
Position			
Pitcher	20 (22.7)		
Fielder	68 (77.3)		
Current injury	22 (27.5)		
Previous injury	33 (37.5)		
Previous surgery	1 (0.01)		

**Table 2:** Absolute values of all scores at the first test (n = 88) J-KJOC: Japanese version of Kerlan–Jobe Orthopedic Clinic questionnaire; Q-DASH: Quick-Disabilities of the Arm, Shoulder, and Hand questionnaire

Questionaire items	Mean (SD)	Range	95% CI
J-KJOC total score	76.9 (17.1)	30–100	72.1–79.5
Item 1	7.2 (2.7)	0–10	6.6–7.7
Item 2	7.3 (2.4)	0.6–10	6.8–7.8
Item 3	7.0 (3.0)	1.2–10	6.4–7.6
Item 4	9.1 (1.6)	0.3–10	8.8–9.4
Item 5	7.4 (2.9)	3.3–10	6.8–8.0
Item 6	7.3 (2.7)	0–10	6.7–7.8
Item 7	7.5 (2.7)	1.8–10	6.9–8.1
Item 8	7.6 (2.6)	0.4–10	7.1–8.2
Item 9	7.8 (1.9)	1.2–10	7.4–8.2
Item 10	7.2 (2.5)	3.2–10	6.7–7.7
Q-DASH score			
Disability/symptom	3.5 (4.7)	0–22.7	2.5–4.5
Work	0.1 (0.9)	0–6.5	-0.1–0.3
Sports/music	10.1 (15.8)	0–81.2	6.7–13.5

Reproducibility and validity

We performed a correlation analysis between the J-KJOC and Q-DASH (disability/symptoms, work, and sports/music) scores of the 88 players (Table 3). The J-KJOC score was correlated with the Q-DASH-disability/symptom (r = -0.60, p<0.01), work (r = -0.11, p = 0.316), and sports/music (r = -0.63, p<0.01) scores. The internal consistency of the J-KJOC was evaluated as excellent (test: α = 0.88), which indicates good homogeneity within the questionnaire regarding the 10 items (Table 3).

**Table 3:** Internal consistency and construct validity of the J-KJOC (n = 88) *Indicates p<0.05 J-KJOC: Japanese version of the Kerlan-Jobe Orthopedic Clinic questionnaire; Q-DASH: Quick Disabilities of the Arm, Shoulder, and Hand questionnaire

J-KJOC total score	Cronbach’s α	Pearson correlation coefficient (r)		
		Q-DASH (disability/symptom)	Q-DASH (work)	Q-DASH (sports/music)
	0.88	−0.60*	−0.11	−0.63*

The median duration of the test-retest score was two weeks. The ICC of the J-KJOC total score was 0.91 (0.40-0.88 for single items, p<0.01) (Table 4). The SEM and MDC of the J-KJOC total score were 4.2 and 1.5, respectively.

**Table 4:** Test-retest and absolute reliability of the J-KJOC (n = 30) J-KJOC: Japanese version of the Kerlan-Jobe Orthopedic Clinic questionnaire; LOA: Limits of agreement; ICC: Intraclass correlation coefficient

J-KJOC	Test (SD)	Retest (SD)	ICC_1.1 _(95% CI)	LOA (95% CI for lower/upper LOA)
Item 1	7.3 (2.6)	8.0 (2.0)	0.64 (0.38–0.81)	
Item 2	7.3 (2.6)	7.1 (2.4)	0.52 (0.20–0.74)	
Item 3	7.8 (2.1)	8.0 (2.1)	0.84 (0.69–0.92)	
Item 4	7.4 (3.6)	7.0 (2.6)	0.78 (0.59–0.89)	
Item 5	9.2 (1.4)	9.3 (1.3)	0.63 (0.35–0.80)	
Item 6	7.1 (2.7)	7.7 (2.7)	0.85 (0.70–0.92)	
Item 7	7.2 (2.7)	7.6 (2.5)	0.88 (0.72–0.94)	
Item 8	8.0 (2.3)	8.5 (1.9)	0.74 (0.53–0.87)	
Item 9	7.8 (2.4)	8.0 (2.4)	0.86 (0.72–0.93)	
Item 10	7.8 (1.7)	7.9 (1.8)	0.55 (0.24–0.76)	
Total score	76.9 (15.8)	79.1 (17.1)	0.91 (0.81–0.95)	−4.8 to 0.5 (−21.4 to −1.5 / −2.8 to 17.1)

**Figure 1:**
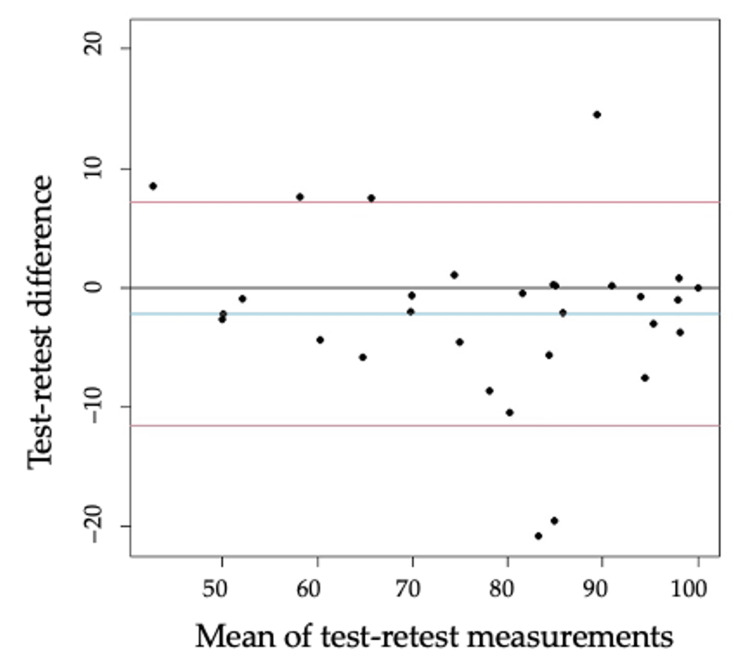
Bland–Altman plot describing the test-retest reliability of the J-KJOC The blue line indicates the mean, the grey line indicates zero, and the red line marks the lower and upper bounds of the 95% CI. J-KJOC: Japanese version of Kerlan-Jobe Orthopedic Clinic questionnaire

The Bland-Altman plot indicates the difference in distribution against the mean between the test and retest scores (Figure 1). A fixed bias did not exist in the J-KJOC scores (mean difference: −2.2; 95% CI: −4.8 to 0.5).

## Discussion

This study demonstrated that the J-KJOC questionnaire is a valid and reliable tool for assessing upper limb performance in Japanese-speaking overhead athletes. A standardized cross-cultural adaptation process is necessary when translating and introducing evaluations into Japan [24]. Furthermore, this method was used to translate the KJOC, indicating the usefulness of this evaluation. Furthermore, a few cultural and linguistic adjustments were required to produce an equivalent to the original questionnaire. Therefore, the J-KJOC is easy to understand for athletes.

Regarding validity, the J-KJOC scores were negatively correlated with the Q-DASH- disability/symptom (r = −0.60, p<0.01) and Q-DASH-sports/music (r = −0.63, p<0.01) scores. Contrastingly, it was not correlated with Q-DASH-work scores. Regarding the correlation with the J-KJOC, Q-DASH-disability/symptom and Q-DASH-work demonstrated lower correlation coefficients than those in a previous study, whereas Q-DASH-sports/music demonstrated correlation coefficients similar to those in a previous study [21]. The participants in that previous study were professional baseball players, whereas those in the present study were university students. In our study, the J-KJOC scores correlated strongly with the Q-DASH-sports/music scores, which is consistent with a previous study, confirming the validity of the J-KJOC as an athlete-specific upper-limb function assessment.

The J-KJOC demonstrated high internal consistency and intra-rater reliability. Its Cronbach's α coefficient was ≥0.7, indicating good internal consistency [26]. In this study, Cronbach's α for the test and retest was 0.88, respectively, similar to the results of previous studies [21,22]. Regarding intra-rater reliability, the criteria for ICC were referenced from previous studies as follows: excellent: 0.75 to 1, good: 0.6 to 0.74, fair: 0.4 to 0.59, and poor: ≤0.4 [27]. The ICC for the total score (0.91) was excellent, indicating that it is a highly reliable assessment method. The Bland-Altman analysis demonstrated no mean difference errors, and the J-KJOC demonstrated consistency with the retest method. Thus, the J-KJOC is a reliable evaluation method.

The ICC should be ideally ≥0.7 as a criterion for good reliability [27]; however, items 1, 2, 5, and 10 did not meet that requirement in our study (Table 4). Item 1 assessed muscle fatigue, item 2 assessed shoulder and elbow pain, and item 10 assessed the effect of arm condition on the competition level, which may depend on the fatigue on the day of assessment. Item 5 assessed the relationship with the team. We targeted amateur athletes and several players who supposedly did not have contracts. This finding is partly attributed to the inclusion of a limited population of collegiate athletes. Older, higher-level athletes are more likely to provide accurate answers about their athletic abilities [22]. Compared with the participants of a previous study, our participants were younger (24.1 in the previous study vs. 20.1 in this study), and they were inferior in terms of athletic history (14.0 in the previous study vs. 11.6 in this study) and athletic level (professional in the previous study vs. collegiate in this study); as such, they may have provided less accurate responses regarding athletic ability [21].

This study has some limitations. First, the measurements were conducted on a limited sample size, which resulted in bias in the players' conditions. Several players without injuries were included in this study (Table 1). Therefore, further research should verify how more severely injured athletes respond to the J-KJOC. Second, we focused on university baseball players without considering other overhead sports or age groups. Thus, our results are inclined toward the function and performance of baseball players. Previous studies included a wide range of athletes from various overhead sports and age groups [16,22]. Moreover, baseline scores for factors contributing to the risk of injury in the KJOC vary across sports [19,20]. Therefore, examining the reliability and validity of the J-KJOC in overhead athletes comprehensively requires a diverse range of sports and age groups. However, the KJOC has been demonstrated to more clearly evaluate function and performance in overhead athletes, unlike conventional upper limb function assessment methods such as Q-DASH, which could be a useful assessment scale for training, rehabilitation, and conditioning plans.

## Conclusions

The J-KJOC questionnaire exhibited good reproducibility and validity in evaluating upper arm function among Japanese university baseball players. Our findings endorse the applicability of the J-KJOC among Japanese-speaking baseball players. Future research should explore its suitability across diverse overhead athlete populations to ascertain its broader relevance in sports medicine applications.

## Figures and Tables

**Figure 2:**
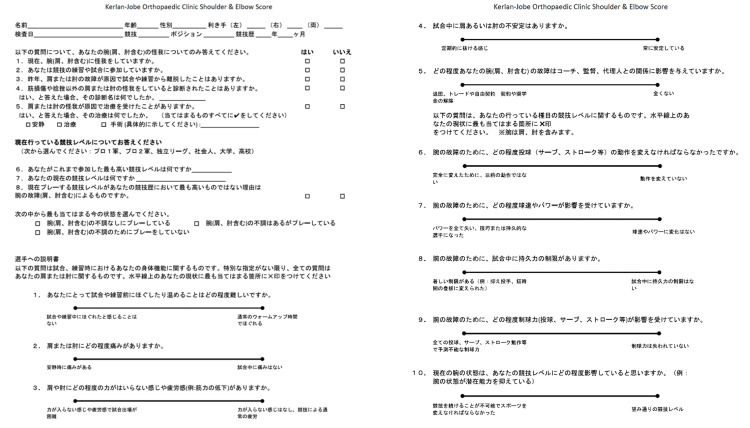
The J-KJOC shoulder and elbow questionnaire J-KJOC: Japanese version of the Kerlan-Jobe Orthopedic Clinic questionnaire

**Figure 3:**
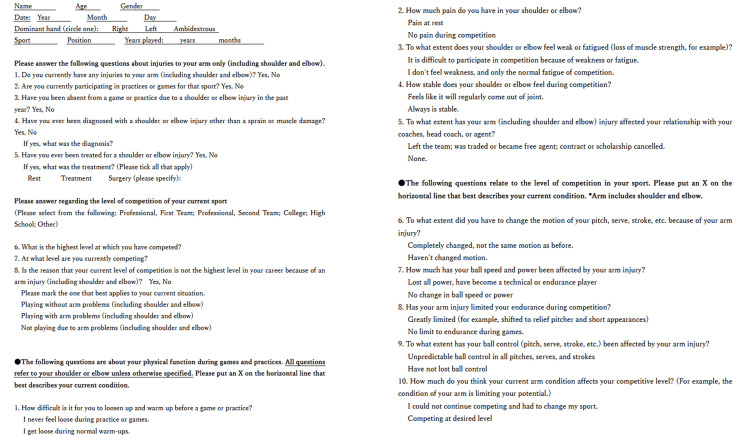
English back-translation of the J-KJOC questionnaire J-KJOC: Japanese version of the Kerlan-Jobe Orthopedic Clinic questionnaire

## Appendices

Appendix A 

Figure 2 features the J-KJOC questionnaire and Figure 3 is the English back-translation of the J-KJOC questionnaire.
